# Giving formulary and drug cost information to providers and impact on medication cost and use: a longitudinal non-randomized study

**DOI:** 10.1186/s12913-016-1752-4

**Published:** 2016-09-21

**Authors:** Chien-Wen Tseng, Grace A. Lin, James Davis, Deborah A. Taira, Jinoos Yazdany, Qimei He, Randi Chen, Allison Imamura, R. Adams Dudley

**Affiliations:** 1 Department of Family Medicine and Community Health, University of Hawaii John A. Burns School of Medicine, 677 Ala Moana Blvd, Ste. 815, Honolulu, HI 96813 USA; 2 Pacific Health Research and Education Institute, Honolulu, USA; 3 Veteran Affairs Pacific Islands Health Care System, Honolulu, USA; 4 Division of General Internal Medicine, Department of Medicine, University of California, San Francisco, USA; 5 Philip R. Lee Institute for Health Policy Studies, University of California, San Francisco, USA; 6 Biostatistics and Data Management Core, University of Hawaii John A. Burns School of Medicine, Honolulu, USA; 7 Daniel K. Inouye College of Pharmacy, University of Hawai’i at Hilo, Hilo, USA; 8 Division of Rheumatology, Department of Medicine, University of California, San Francisco, USA; 9 Library Business Services, University of California, Los Angeles, USA; 10 Division of Pulmonary and Critical Care, Department of Medicine, University of California, San Francisco, USA

**Keywords:** Prescription drug costs, Out-of-pocket cost, Formularies, Medication use, Electronic prescribing

## Abstract

**Background:**

Providers wish to help patients with prescription costs but often lack drug cost information. We examined whether giving providers formulary and drug cost information was associated with changes in their diabetes patients’ drug costs and use. We conducted a longitudinal non-randomized evaluation of the web-based Prescribing Guide (www.PrescribingGuide.com), a free resource available to Hawaii’s providers since 2006, which summarizes the formularies and copayments of six health plans for drugs to treat 16 common health conditions. All adult primary care physicians in Hawaii were offered the Prescribing Guide, and providers who enrolled received a link to the website and regular hardcopy updates.

**Methods:**

We analyzed prescription claims from a large health plan in Hawaii for 5,883 members with diabetes from 2007 (baseline) to 2009 (follow-up). Patients were linked to 299 “main prescribing” providers, who on average, accounted for >88 % of patients’ prescriptions and drug costs. We compared changes in drug costs and use for “study” patients whose main provider enrolled to receive the Prescribing Guide, versus “control” patients whose main provider did not enroll to receive the Prescribing Guide.

**Results:**

In multivariate analyses controlling for provider specialty and clustering of patients by providers, both patient groups experienced similar increases in number of prescriptions (+3.2 vs. +2.7 increase, *p* = 0.24), and days supply of medications (+141 vs. +129 increase, *p* = 0.40) averaged across all drugs. Total and out-of-pocket drug costs also increased for both control and study patients. However, control patients showed higher increases in yearly total drug costs of $208 per patient (+$792 vs. +$584 increase, *p* = 0.02) and in 30-day supply costs (+$9.40 vs. +$6.08 increase, *p* = 0.03). Both groups experienced similar changes in yearly out-of-pocket costs (+$41 vs + $31 increase, *p* = 0.36) and per 30-day supply (−$0.23 vs. −$0.19 decrease, *p* = 0.996).

**Conclusion:**

Giving formulary and drug cost information to providers was associated with lower increases in total drug costs but not with lower out-of-pocket costs or greater medication use. Insurers and health information technology businesses should continue to increase providers’ access to formulary and drug cost information at the point of care.

**Electronic supplementary material:**

The online version of this article (doi:10.1186/s12913-016-1752-4) contains supplementary material, which is available to authorized users.

## Background

Drug costs in the United States are rapidly rising and providers and patients are increasingly being asked to be aware of medication cost when choosing treatments [1–3]. At the same time, drug benefits have become more complex, with increasing number of coverage tiers and different cost-sharing requirements for each tier [4]. Therefore, providers’ and patients’ choice of which prescription drugs to use can substantially affect patients’ out-of-pocket costs and even medication adherence [5]. A study of 1.1 million insured persons found that nearly half of patients could potentially switch to lower cost but potentially effective drugs within the same treatment class, decreasing total drug costs between $389 and $452 per person and decreasing out-of-pocket costs by $22 to $113 per person annually [6].

Currently, there is a need for better coordination to give formulary and drug cost information to providers and patients at the point of care, such as in the office setting [7, 8]. Although providers are willing to help their patients by prescribing less expensive drugs if appropriate and available [9, 10], few providers accurately know such cost information [10–12]. In our statewide survey of 247 adult primary care physicians, nearly 100 % wanted to help patients with drug costs, but 9 in 10 said that difficulty knowing cost information prevented them from doing so [12]. This is because providers often contract with multiple plans, up to 10 or more, and formularies and copayments for the same drug may vary between plans as well as over time [13–15]. While health plans make formulary and drug cost information available on their websites, such data may not be linked to e-prescribing software [16]. In an industry survey of 200 physicians, fewer than half had access to formularies with their e-prescribing software, and fewer than one-third had access to prior authorization or copayment information [16]. Lack of easy access to formulary and drug cost information for providers can result in their overlooking potentially effective, lower cost drugs [6]. At the same time, studies show that giving providers formulary decision support can increase rates of prescribing drugs covered by formularies and drugs with lower tier copayments [17–23].

**Fig. 1:**
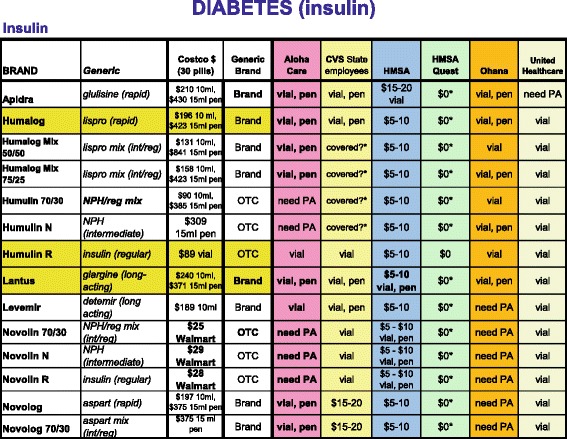
Prescribing Guide – Diabetes. An excerpt from the Prescribing Guide showing formulary coverage and copayment information across six health plans for insulin drugs used to treat diabetes

We designed a community intervention to give providers improved access to formulary and drug cost information and measured changes in their patients’ drug costs and use. Since 2006, we have provided a free web-based “**Prescribing Guide**” **(PG)** as a clinical resource for Hawaii’s providers. The PG summarizes formulary coverage and copayments from six health plans for drugs used to treat 16 common health conditions (e.g. asthma, diabetes, hypertension, etc.). For each treatment class, the PG describes which drugs in that class are covered, which drugs are brand-name or generic, preferred or non-preferred, which require prior authorizations, and the approximate copayments charged by each plan (Fig. 1). The PG also highlights which drugs in each class are widely covered at lower copayments by all six plans, helping providers to learn quickly which drugs are less expensive and likely to be covered for patients. Initially, a hardcopy of the PG was mailed to all adult primary care providers in the state identified from the Hawaii Medical Association’s list of all licensed Hawaii physicians. Half of providers (56 %) voluntarily signed up to continue receiving quarterly PG updates. Shortly thereafter, a web-based version www.PrescribingGuide.com was also developed and the website link was sent to those providers who enrolled, to use as needed with no active reminders in their workflow. Providers were surveyed annually to confirm that they still wished to receive the PG and that they were still using this resource. The one-year follow-up survey indicated that the PG doubled the percentage of providers who reported checking formularies (34 to 67 %) and knew drug costs (11 to 29 %). The PG is inexpensive; maintaining and updating the website for providers statewide now costs less than $5,000 per year.

In this study, we examined the changes in drug costs and medication use for study patients whose providers received the PG versus control patients whose providers did not receive the PG. If giving better access to formulary and drug cost information to providers lowers their patients’ drug costs and increases medication use, this would support collaborations between insurers and the health information technology industry to make formulary and drug cost information available at the point of care.

## Methods

### Patients

We partnered with a large health plan in Hawaii that covers ~70 % of the state’s privately insured residents [24]. Claims and enrollment data were used to include patients with diabetes in 2007 and 2009 who were enrolled for >320 days in each year and filled one or more oral diabetes prescriptions. Individuals with diabetes were chosen since the great majority of persons with diabetes require medications to reach glycemic control (85 %), are on multiple medications, and are vulnerable to drug costs [25–29]. Between 14 and 49 % of persons with diabetes report medication nonadherence due to cost [26–29] The health plan flagged patients as having diabetes if they had two or more outpatient visits for diabetes based on icd-9 codes (International Statistical Classification of Diseases and Related Health Problems) or one visit for diabetes and one or more prescriptions for a diabetes medication. Medicaid members were excluded since they have $0 prescription copayments. Medicare members were excluded since Part D plans were not included in the PG. During the study period, the health plan made no major changes to their drug benefit design and most commercial members paid tiered dollar copayments for preferred generic and brand-name drugs.

### Study assignment

Each patient was linked to their “main” prescriber, defined as the provider who wrote the greatest number of prescriptions for them in that year. Providers were required to be a general internist, family physician, general practitioner, endocrinologist, or cardiologist and the main prescriber for ten or more patients each year. Patients were eligible if they were linked to the same main prescribing provider in 2007 and 2009. These patients were assigned to the “study” group if their main prescriber enrolled to receive the PG or to the “control” group if their main prescriber did not enroll to receive the PG.

### Medication use and drug costs

For each patient, we calculated the changes from 2007 to 2009 in medication use (number of prescriptions, total days supply of medications), and total drug costs (paid by plan and patient) and patients’ out-of-pocket costs per year and per 30-day supply of medications. These were calculated for all drugs (including non-diabetes medications), and then separately for brand-name versus generic drugs.

### Analyses

We conducted multivariate analyses using SAS 9.4 Proc Mixed to determine whether control patients and study patients were different at baseline (2007) and follow-up (2009) in terms of medication use and drug costs [30]. To examine the impact of the PG, we tested for differences in the *changes* in medication use and drug costs over time for control patients vs. study patients. The Proc Mixed procedure was chosen because it is fairly robust, does not rely on the dependent variables (e.g. medication use, drug costs) being normally distributed, and can account for patient clustering by provider (i.e. correlation in outcomes between patients with the same main prescribing provider) [30]. For all analyses, the main predictor was whether the patient’s provider received the PG (study patients) or not (control patients) and we controlled for provider specialty. We also conducted sensitivity analyses restricting analyses to primary care providers and to drug claims from the main prescribing providers, which did not change the main study findings.

## Results

**Fig. 2:**
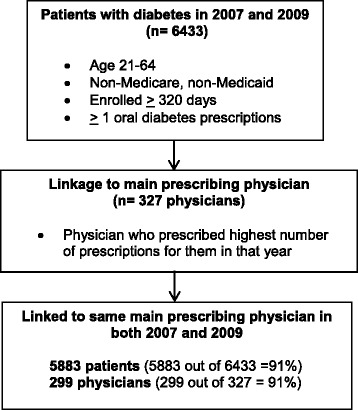
Patient eligibility and linkage to main prescribing physician. A flowchart showing the link between eligible plan enrollees with diabetes and their main prescribing physician who prescribed the highest number of prescriptions for them in a given year

A total of 6,433 patients ages 21–64 with diabetes were enrolled for ≥320 days and filled at least one oral diabetes prescription in both 2007 and 2009. These patients were linked to 327 “main prescribing” providers who prescribed the highest number of prescriptions for them in either 2007 or 2009 (Fig. 2). Of these patients, 5,883 (91 %) were linked to the same “main prescribing” provider in both years (299 providers) and included in our final analyses. Linkage was tight, with main prescribing providers accounting for the vast majority of their patients’ number of prescriptions (88 %), days supply of medications (90 %), total drug costs (89 %), and out-of-pocket costs (88 %). Providers were mainly general internists (69 %), family physicians (17 %), and endocrinologists (8 %), followed by general practitioners (5 %) and cardiologists (1 %).

The 5,883 patients represented $42.7 million in total drug costs, $5.96 million in out-of-pocket drug costs, 433,945 prescriptions, and 15.3 million days supply of medications for the two years.

At baseline, both control and study patients started with similar yearly overall medication use, total drug costs, and out-of-pocket costs based on all drugs. Both groups began with comparable number of prescriptions per year (35.5 vs. 35.4, *p* = 0.48), days supply of medications (1233 vs. 1233, *p* = 0.34), yearly total drug costs ($3340 vs. $3216, p = 0.74), and yearly out-of-pocket costs ($503 vs. $473, *p* = 0.39) (Table 1). However, at baseline, control patients used less of generic drugs in terms of number of generic prescriptions (19.9 vs. 21.1, *p* = 0.02) and days supply of generic drugs (675 vs 727, *p* = .003). Thus, control patients started with higher total drug costs per 30-day supply ($81 vs. $77, *p* = 0.03) as well as higher out-of-pocket costs per 30-day supply ($12 vs. $11, *p* = 0.01) than did study patients. Standard deviations for Basline medication use and cost in 2007 (Table 1) are available in Additional file 1: Appendix 1.

**Table 1:** BASELINE medication use and cost in 2007^a^

	All drugs			Generic drugs			Brand-name drugs		
Baseline year (2007)	Control	Study	p-value	Control	Study	p-value	Control	Study	p-value
Medication use									
Number of prescriptions	35.5	35.4	0.48	19.9	21.1	0.02*	15.6	14.3	0.13
Total days supply	1233	1233	0.34	675	727	0.003*	558	506	0.11
Total drug cost, $									
Per year	3340	3216	0.74	860	925	0.02*	2480	2291	0.29
Per 30-day supply	81	77	0.03*	40	40	0.51	129	127	0.55
Out-of-pocket cost, $									
Per year	503	473	0.40	112	120	0.005*	391	353	0.15
Per 30-day supply	12	11	0.01*	5	5	0.11	21	20	0.22

*Statistically significant at *p* < 0.05

^a^Multivariate analyses SAS Proc Mixed 9.4 comparing Control (*n* = 3061) vs. Study patients (*n* = 2822), controlling for clustering by provider and controlling for provider specialty. Standard deviations for Baseline medication use and cost in 2007 are available in Additional file 1: Appendix 1.

At follow-up in 2009, control patients continued to have both higher total drug costs per 30-day supply ($90 vs. $83, *p* = 0.003) as well as higher out-of-pocket costs per 30-day supply ($12 vs. $11, *p* = .01). Again, this was due to control patients’ trend of using less of generic drugs in terms of number of generic prescriptions (22.0 vs. 23.0, *p* = 0.08) and days supply of generic drugs (768 vs. 815, *p* = 0.02) (Table 2). However, control patients now also used more brand-name drugs in terms of yearly number of brand-name prescriptions (16.6 vs. 15.0, *p* = 0.04) and days supply of brand-name prescriptions (606 vs. 547, *p* = 0.049), compared to study patients. Standard deviations for Follow-up medication use and cost in 2009 (Table 2) are available in Additional file 1: Appendix 2.

**Table 2:** FOLLOW-UP medication use and cost in 2009^a^

	All drugs			Generic drugs			Brand-name drugs		
Follow-up Year (2009)	Control	Study	p-value	Control	Study	p-value	Control	Study	p-value
Medication use									
Number of prescriptions	38.6	38.0	0.97	22.0	23.0	0.08	16.6	15.0	0.04*
Total days supply	1374	1362	0.67	768	815	0.02*	606	547	0.049*
Total drug cost, $									
Per year	4131	3800	0.11	861	890	0.29	3270	2910	0.049*
Per 30-day supply	90	83	0.003*	36	34	0.02*	159	152	0.08
Out-of-pocket cost, $									
Per year	545	504	0.18	127	134	0.03*	418	370	0.07
Per 30-day supply	12	11	0.01*	6	5	0.02*	21	20	0.03*

*Statistically significant at *p* < 0.05

^a^Multivariate analyses SAS Proc Mixed 9.4 comparing Control (*n* = 3061) vs. Study patients (*n* = 2822), controlling for clustering by provider and controlling for provider specialty. Standard deviations for Follow-up medication use and cost in 2009 are available in Additional file 1: Appendix 2.

In evaluating the impact of the PG by examining the differences in *changes* over time in medication use and drug costs for control versus study patients, both groups experienced comparable increases in overall medication use, brand-name drug use, and generic drug use. Both groups showed similar increases in number of prescriptions (+3.2 vs. +2.7 increase, *p* = 0.24), and in days supply of medications per year (+141 vs. +129 increase, *p* = 0.40) (Table 3). Standard deviations for Change in medication use and cost from Baseline to Follow-up year (Table 3) are available in Additional file 1: Appendix 3.

**Table 3:** CHANGE in medication use and cost from BASELINE to FOLLOW-UP year^a^

	All drugs			Generic drugs			Brand-name drugs		
Change from 2007 to 2009	Control	Study	p-value	Control	Study	p-value	Control	Study	p-value
Medication use									
Number of prescriptions	3.2	2.7	0.24	2.1	1.9	0.43	1.1	0.8	0.32
Total days supply	141	129	0.40	93	89	0.63	48	41	0.45
Total drug cost, $									
Per year	792	584	0.02*	2	−34	0.053	790	619	0.07
Per 30-day supply	9.40	6.08	0.03*	−3.42	−5.11	0.065	30	25	0.20
Out-of-pocket cost, $									
Per year	41	31	0.36	15	14	0.44	26	17	0.44
Per 30-day supply	−0.23	−0.19	0.996	0.30	0.13	0.19	0.32	−0.23	0.27

*Statistically significant at *p* < 0.05

^a^Multivariate analyses SAS Proc Mixed 9.4 comparing Control (*n* = 3061) vs. Study patients (*n* = 2822), controlling for clustering by provider and controlling for provider specialty. Standard deviations for Change in medication use and cost from Baseline to Follow-up year are available in Additional file 1: Appendix 3.

However with respect to cost, increases in yearly total drug cost were $208 higher per control patient (+$792 vs. +$584 increase, *p* = 0.02) than per study patient (Table 3). This higher increase in yearly total drug costs occurred both for generic drugs (+$2 increase vs −$34 decrease, *p* = 0.053) and for brand-name drugs (+$790 vs + $619 increase, *p* = 0.07), both of which approached statistical significance. The higher increase in total drug cost occurred although control patients did not have greater increases in number of prescriptions for brand-name drugs (+1.1 vs. +0.8 increase, *p* = 0.32) and generic drugs (+2.1 vs. +1.9 increase, *p* = 0.43), compared to study patients. Rather for the control group, the cost of generic drugs per 30-day supply tended to drop slower (−$3.42 vs. −$5.11, *p* = 0.065) and the cost of brand-name drugs per 30-day supply tended to rise faster over time (+$30 vs. +$25 increase, *p* = 0.20) than it did for study patients.

With respect to copayments, there were no significant differences between control and study patients in changes in yearly out-of-pocket costs (+$41 vs + $31 increase, *p* = 0.36) or out-of-pocket costs per 30-day supply (−$0.23 vs. −$0.19 decrease, *p* = 0.996).

## Discussion

We found that improving providers’ access to formulary and drug cost information was associated with lower increases in yearly total drug costs averaging $208 per patient, but not lower out-of-pocket costs or increased medication use. Since out-of-pocket costs were not affected, this represents primarily savings to health plans rather than direct savings for patients. However, there may be indirect benefits to patients if lower total drug costs for insurers lead to fewer premium increases. Our study used a simple website to provide such drug cost information, but our results support findings from the limited number of larger scale studies examining the integration of formularies and drug costs into e-prescribing [18–21]. Fischer’s study of e-prescribing with formulary support for 1.5 million patients estimated total drug cost savings of $845,000 per 100,000 patients assuming a 20 % uptake among their providers [18]. McMullin’s study of 38 primary care providers with half receiving e-prescribing with clinical decision support (including preferred drug options) estimated total drug cost savings at $1.07 per member per month (~$1.2 million per 100,000 patients) [19, 20]. Zuker’s study of 647 providers and e-prescribing with formulary support estimated total drug cost savings of 4 % [21]. These studies did not report whether giving formulary information reduced out-of-pocket costs or increased medication use. Our study did not find such an impact. However, Pevnick’s study of 297 providers and e-prescribing with formulary support estimated modest decreases in out-of-pocket costs when focusing on two drug classes: angiotensin-receptor blockers and inhaled steroids, although there was no increase in their medication use (adherence) [17]. Overall, our study supports the hypothesis that giving providers formulary and drug cost information can potentially lower total drug costs.

We found that both groups had similar increases in their use of brand-name and generic drugs (number of prescriptions, days supply of medication). This indicates that the savings in total drug costs came surprisingly, not from greater use of generic versus brand-name drugs (e.g. switching from brand-name to generic drugs or new prescriptions being written for generic rather than brand-name drugs). Also, changes in out-of-pocket costs were similar, meaning the total drug cost savings did not come from choosing formulary versus non-formulary drugs, or from choosing lower tiered drugs which would have led to lower out-of-pocket costs. This is surprising since prior studies show that giving formulary and drug cost information to providers increases their prescribing of generic and formulary preferred drugs [17–23]. In our study, the lower increase in yearly total drug costs were in part due to the study group experiencing slower increases in total drug costs per 30-day supply for their brand-name and generic drugs. This is unexpected since the PG gave providers formulary information and out-of-pocket costs, and not total drug costs. The PG may have had this effect because we intentionally highlighted “widely covered” drugs (bolded with yellow highlights) so that providers could easily learn which drugs were likely to be covered and low cost for their patients regardless of the drug plan. Prescribing these widely covered drugs could decrease total drug costs if health plans preferentially cover these drugs because they are less expensive for insurers to purchase. Thus when giving providers formulary and drug cost information, consideration should be given to highlight which drugs are widely covered by all plans at lower cost.

Similar to prior studies, ours was not a randomized control trial [17–23]. We also had access only to prescription claims data and could not control for important patient-level characteristics such as age, gender, and co-morbidities which can impact drug costs and adherence [31]. However, prior to the PG intervention, both study and control patients started with similar overall medication use (total number of prescriptions, days supply of medication, and days supply per prescription) and overall drug costs (yearly total drug costs and yearly out-of-pocket costs) although study patients did start with higher generic drug use. Also, over half of all the adult primary care providers in Hawaii who were offered the PG voluntarily signed up to continue receiving updates, indicating the substantial interest from providers to have better access to formulary and drug information even in low-technology form. Thus, our findings should lend support to greater efforts to give formulary and drug cost information to providers and patients. This is especially important since the current trend is for drug benefits to become increasingly more complex with growing number of coverage tiers and copayments [4, 5].

Our PG had the advantage of easy development (over a few months), being free to providers, pharmacists, and office staff, and requiring no proprietary software or user licenses for use. Pharmacy students now maintain the PG website for the entire state at minimal cost by checking formularies each month. However, on a national level, a more practical approach would be to increase the linkage of formularies and drug costs to e-prescribing [32–34]. With the Affordable Care Act, providers face a 1.5 % payment reduction in reimbursements unless they adopt e-prescribing [35]. In 2014 e-prescribing increased to 70 % of providers, up from 7 % in 2008 [36]. Greater linkage of formulary and copayment data with e-prescribing software would be invaluable to giving providers and patients access to drug cost information at the point of care.

A major limitation of our study was that it was not randomized, and our analyses could not include patient characteristics which can affect medication use such as age, race, sex, income which were not available from prescription claims [31]. We were able to control for provider specialty, but did not have access to other provider characteristics, such as number of years in practice, which may affect knowledge of drug costs. The study focused on patients with diabetes with at least one oral medication, although all drugs were include in analyses, and the PG may have less impact on drug costs for healthier patients or those who are on fewer medications. Lastly, unlike e-prescribing, we were unable to measure PG use directly. However, we conducted annual follow-up written questionnaires and telephone calls to confirm through providers’ self-report that they were still using the PG.

## Conclusion

Giving providers formulary and drug cost information at the point of care was associated with lower increases in annual total drug costs without significant impact on out-of-pocket costs or medication use. On a national level, insurers such as Medicare, Medicaid, and commercial health plans should continue to partner with health information technology businesses to improve providers’ and patients’ access to formulary and drug cost information at the point of care.

## Additional file

Additional file 1: Appendix 1. BASELINE medication use and cost in 2007 – Means and standard deviations. Baseline medication use and cost of patients’ prescription drugs in 2007. Appendix 1 is the same as Table 1 in the manuscript, but includes the standard deviations. Appendix 2. FOLLOW-UP medication use and cost in 2009 – Means and standard deviations. Follow-up medication use and cost of patients’ prescription drugs in 2009. Appendix 2 is the same as Table 2 in the manuscript, but includes the standard deviations. Appendix 3. CHANGE in medication use and cost from BASELINE to FOLLOW-UP year – Means and standard deviations. Change in medication use and cost of patients’ prescription drugs from 2007 to 2009. Appendix 3 is the same as Table 3 in the manuscript, but includes the standard deviations. (DOCX 22 kb)

## Abbreviations

PG: Prescribing Guide
