# Supplementary material for: Giving formulary and drug cost information to providers and impact on medication cost and use: a longitudinal non-randomized study
Source: BMC Health Serv Res. 2016 Sep 21;16:499. doi: 10.1186/s12913-016-1752-4 (PMC5031286; doi:10.1186/s12913-016-1752-4)
Supplement: Additional file 1: Appendix 1. — BASELINE medication use and cost in 2007 – Means and standard deviations. Baseline medication use and cost of patients’ prescription drugs in 2007. Appendix 1 is the same as Table 1 in the manuscript, but includes the standard deviations. Appendix 2. FOLLOW-UP medication use and cost in 2009 – Means and standard deviations. Follow-up medication use and cost of patients’ prescription drugs in 2009. Appendix 2 is the same as Table 2 in the manuscript, but includes the standard deviations. Appendix 3. CHANGE in medication use and cost from BASELINE to FOLLOW-UP year – Means and standard deviations. Change in medication use and cost of patients’ prescription drugs from 2007 to 2009. Appendix 3 is the same as Table 3 in the manuscript, but includes the standard deviations. (DOCX 22 kb) [file 12913_2016_1752_MOESM1_ESM.docx]

**Appendix 1. BASELINE medication use and cost in 2007 – Means and standard deviations**

|  |  |  | **All drugs** | | |  |  | **Generic drugs** | | |  |  | **Brand-name drugs** | | |  |
| --- | --- | --- | --- | --- | --- | --- | --- | --- | --- | --- | --- | --- | --- | --- | --- | --- |
| **Baseline year (2007)*** | |  | **Control** | **Study** | **p-**  **value** |  |  | **Control** | **Study** | **p-value** |  |  | **Control** | **Study** | **p-value** |  |
| Medication use (SD) | |  |  |  |  |  |  |  |  |  |  |  |  |  |  |  |
|  | Number of prescriptions | | 35.5 (23.6) | 35.4 (23.5) | 0.48 |  |  | 19.9 (15.3) | 21.1 (15.6) | 0.02** |  |  | 15.6 (14.3) | 14.3 (14.3) | 0.13 |  |
|  | Total days supply |  | 1233 (773) | 1233 (760) | 0.34 |  |  | 675 (501) | 727 (511) | 0.003** |  |  | 558 (491) | 506 (472) | 0.11 |  |
| Total drug cost, $ (SD) | | |  |  |  |  |  |  |  |  |  |  |  |  |  |  |
|  | Per year |  | 3340 (2650) | 3216 (2794) | 0.74 |  |  | 860 (940) | 925 (755) | 0.02** |  |  | 2480 (2317) | 2291 (2556) | 0.29 |  |
|  | Per 30-day supply |  | 81 (37) | 77 (42) | 0.03** |  |  | 40 (26) | 40 (26) | 0.51 |  |  | 129 (74) | 127 (101) | 0.55 |  |
| Out-of-pocket cost, $ (SD) | | | |  |  |  |  |  |  |  |  |  |  |  |  |  |
|  | Per year |  | 503 (491) | 473 (435) | 0.40 |  |  | 112 (99) | 120 (104) | 0.005** |  |  | 391 (453) | 353 (398) | 0.15 |  |
|  | Per 30-day supply |  | 12 (8) | 11 (7) | 0.01** |  |  | 5 (4) | 5 (3) | 0.11 |  |  | 21 (16) | 20 (21) | 0.22 |  |
| *Multivariate analyses SAS Proc Mixed 9.4 comparing Control (n= 3061) vs. Study patients (n=2822), controlling for clustering by provider and controlling for provider specialty | | | | | | | | | | | | | | | |  |
| **Statistically significant at p < 0.05 | | | | | | | | | | | | | | | |  |

**Appendix 2. FOLLOW-UP medication use and cost in 2009 – Means and standard deviations**

|  |  |  | **All drugs** | | |  |  | **Generic drugs** | | |  |  | **Brand-name drugs** | | |
| --- | --- | --- | --- | --- | --- | --- | --- | --- | --- | --- | --- | --- | --- | --- | --- |
| **Follow-up Year (2009)*** | |  | **Control** | **Study** | **p-**  **value** |  |  | **Control** | **Study** | **p-value** |  |  | **Control** | **Study** | **p-value** |
| Medication use (SD) | |  |  |  |  |  |  |  |  |  |  |  |  |  |  |
|  | Number of  prescriptions | | 38.6 (24.4) | 38.0 (24.0) | 0.97 |  |  | 22.0 (16.5) | 23.0 (16.7) | 0.08 |  |  | 16.6 (14.5) | 15.0 (14.6) | 0.04** |
|  | Total days supply |  | 1374 (792) | 1362 (764) | 0.67 |  |  | 768 (537) | 815 (538) | 0.02** |  |  | 606 (505) | 547 (489) | 0.049** |
| Total drug cost, $ (SD) | |  |  |  |  |  |  |  |  |  |  |  |  |  |  |
|  | Per year |  | 4131 (3293) | 3800 (3096) | 0.11 |  |  | 861 (801) | 890 (738) | 0.29 |  |  | 3270 (3039) | 2910 (2885) | 0.049** |
|  | Per 30-day supply |  | 90 (47) | 83 (46) | 0.003** |  |  | 36 (30) | 34 (23) | 0.02** |  |  | 159 (160) | 152 (101) | 0.08 |
| Out-of-pocket cost, $ | |  |  |  |  |  |  |  |  |  |  |  |  |  |  |
|  | Per year |  | 545 (554) | 504 (439) | 0.18 |  |  | 127 (103) | 134 (115) | 0.03** |  |  | 418 (519) | 370 (409) | 0.07 |
|  | Per 30-day supply |  | 12 (7) | 11 (7) | 0.01** |  |  | 6 (7) | 5 (5) | 0.02** |  |  | 21 (22) | 20 (17) | 0.03** |
| *Multivariate analyses SAS Proc Mixed 9.4 comparing Control (n= 3061) vs. Study patients (n=2822), controlling for clustering by provider and controlling for provider specialty | | | | | | | | | | | | | | | |
| **Statistically significant at p < 0.05 | | | | | | | | | | | | | | | |

**Appendix 3. CHANGE in medication use and cost from BASELINE to FOLLOW-UP year – Means and standard deviations.**

|  |  | **All drugs** | | | **Generic drugs** | | |  | **Brand-name drugs** | | |
| --- | --- | --- | --- | --- | --- | --- | --- | --- | --- | --- | --- |
| **Change from 2007 to 2009*** | | **Control** | **Study** | **p-value** | **Control** | **Study** | **p-value** |  | **Control** | **Study** | **p-value** |
| Medication use (SD) | | | | | | | | | | | |
| Number of  prescriptions | | 3.2 (16.4) | 2.7 (16.3) | 0.24 | 2.1 (11.6) | 1.9 (12.0) | 0.43 |  | 1.1 (10.4) | 0.8 (9.7) | 0.32 |
| Total days supply | | 141 (509) | 129 (502) | 0.40 | 93 (369) | 89 (371) | 0.63 |  | 48 (345) | 41 (321) | 0.45 |
| Total drug cost, $ (SD) | | | | | | | | | | | |
| Per year | | 792 (2260) | 584 (2116) | 0.02** | 2 (651) | -34 (610) | 0.053 |  | 790 (2152) | 619 (2017) | 0.07 |
| Per 30-day supply | | 9.40 (39) | 6.08 (34) | 0.03** | -3.42 (35) | -5.11 (24) | 0.065 |  | 30 (82) | 25 (111) | 0.20 |
| Out-of-pocket cost, $ (SD) | | | | | | | | | | | |
| Per year | | 41 (366) | 31 (321) | 0.36 | 15 (74) | 14 (77) | 0.44 |  | 26 (351) | 17 (301) | 0.44 |
| Per 30-day supply | | -0.23 (6) | -0.19 (5) | 0.996 | 0.30 (8) | 0.13 (5) | 0.19 |  | 0.32 (25) | -0.23 (22) | 0.27 |
| *Multivariate analyses SAS Proc Mixed 9.4 comparing Control (n= 3061) vs. Study patients (n=2822), controlling for clustering by provider and controlling for provider specialty | | | | | | | | | | | |
| **Statistically significant at p < 0.05 | | | | | | | | | | | |
